# Identification of Pyroptosis-Relevant Signature in Tumor Immune Microenvironment and Prognosis in Skin Cutaneous Melanoma Using Network Analysis

**DOI:** 10.1155/2023/3827999

**Published:** 2023-02-08

**Authors:** Yun Zhu, Dan Han, Hongjue Duan, Qi Rao, Yike Qian, Qiaoyun Chen, Xiao Du, Huanyu Ni, Siliang Wang

**Affiliations:** ^1^Department of Pharmacy, Nanjing Drum Tower Hospital, The Affiliated Hospital of Nanjing University Medical School, Nanjing, China; ^2^Nanjing Medical Center for Clinical Pharmacy, Nanjing 210008, China; ^3^School of Basic Medicine and Clinical Pharmacy, China Pharmaceutical University, Nanjing 211198, China

## Abstract

**Background:**

Pyroptosis is closely related to the programmed death of cancer cells as well as the tumor immune microenvironment (TIME) via the host-tumor crosstalk. However, the role of pyroptosis-related genes as prognosis and TIME-related biomarkers in skin cutaneous melanoma (SKCM) patients remains unknown.

**Methods:**

We evaluated the expression profiles, copy number variations, and somatic mutations (CNVs) of 27 genes obtained from MSigDB database regulating pyroptosis among TCGA-SKCM patients. Thereafter, we conducted single-sample gene set enrichment analysis (ssGSEA) for evaluating pyroptosis-associated expression patterns among cases and for exploring the associations with clinicopathological factors and prognostic outcome. In addition, a prognostic pyroptosis-related signature (PPRS) model was constructed by performing Cox regression, weighted gene coexpression network analysis (WGCNA), and least absolute shrinkage and selection operator (LASSO) analysis to score SKCM patients. On the other hand, we plotted the ROC and survival curves for model evaluation and verified the robustness of the model through external test sets (GSE22153, GSE54467, and GSE65904). Meanwhile, we examined the relations of clinical characteristics, oncogene mutations, biological processes (BPs), tumor stemness, immune infiltration degrees, immune checkpoints (ICs), and treatment response with PPRS via multiple methods, including immunophenoscore (IPS) analysis, gene set variation analysis (GSVA), ESTIMATE, and CIBERSORT. Finally, we constructed a nomogram incorporating PPRS and clinical characteristics to improve risk evaluation of SKCM.

**Results:**

Many pyroptosis-regulated genes showed abnormal expression within SKCM. TP53, TP63, IL1B, IL18, IRF2, CASP5, CHMP4C, CHMP7, CASP1, and GSDME were detected with somatic mutations, among which, a majority displayed CNVs at high frequencies. Pyroptosis-associated profiles established based on pyroptosis-regulated genes showed markedly negative relation to low stage and superior prognostic outcome. Blue module was found to be highly positively correlated with pyroptosis. Later, this study established PPRS based on the expression of 8 PAGs (namely, GBP2, HPDL, FCGR2A, IFITM1, HAPLN3, CCL8, TRIM34, and GRIPAP1), which was highly associated with OS, oncogene mutations, tumor stemness, immune infiltration degrees, IC levels, treatment responses, and multiple biological processes (including cell cycle and immunoinflammatory response) in training and test set samples.

**Conclusions:**

Based on our observations, analyzing modification patterns associated with pyroptosis among diverse cancer samples via PPRS is important, which can provide more insights into TIME infiltration features and facilitate immunotherapeutic development as well as prognosis prediction.

## 1. Introduction

Melanoma represents a deadly skin tumor with high aggressiveness, which takes up around 4% of skin cancer cases, with a high mortality rate of 80% [1]. Melanoma will invade the melanocyte-containing tissues, in particular the skin [2]. Recently, melanoma is mainly treated with traditional chemotherapy, radiotherapy, and treatments that target B-raf proto-oncogene and MAP kinase-ERK kinase [3]. The 5-year survival of primary SKCM is as high as 95%, but that is <10% in metastatic SKCM patients because of the lacking effective biomarkers and high relapse rate. Therefore, examining molecular changes and investigating the carcinogenesis- and prognosis-related molecular mechanisms of SKCM prognosis is of great importance, which contributes to the development of novel therapy.

Currently, immunotherapy is becoming an effective approach to improve the survival of some solid tumors [4]. It is superior to traditional antitumor treatment modalities, as revealed in diverse experimental and clinical studies, thus contributing to enhancing the disease survival [5]. Immune checkpoint inhibitors (ICIs) have prolonged of survival of patients with metastatic melanoma. Pembrolizumab and nivolumab, the antibodies against programmed cell death protein (PD-1), together with ipilimumab, the antibody against cytotoxic T-lymphocyte-associated protein 4 (CTLA-4), have been extensively adopted in immunotherapy for SKCM [6, 7]. Although great progresses have been achieved in immunotherapy, a considerable portion of SKCM cases still can not respond or develop resistance to PD-1 inhibitors [8]. The following causes may be responsible for the development of resistance, including lacking PD-1 receptors, lacking CD8^+^ T-cells related to endogenously activated WNT-*β*-catenin pathway within melanoma cells, genetic mutations, tumor antigen presentation and mutation, dynamic alterations in tumor immune microenvironment (TIME), and epigenetic alterations of some critical cancer proteins [9]. Consequently, identifying the efficient biomarkers for predicting immunotherapeutic response in melanoma is becoming the novel research direction. To take an example, it is suggested that immune biomarkers including PD-L1 expression, infiltration of CD8^+^ T-cells, copy number variations (CNVs), and somatic mutation burdens contribute to the immunotherapy of SKCM [10–12]. However, there are problems to be settled down. At present, the differences in PD-L1 identification, assessment platforms, and systems may lead to different positive critical values, making it more difficult to form the standard for measuring PD-L1 in tumor cells [13]. Meanwhile, the survival of SKCM has been greatly prolonged thanks to early diagnosis by corresponding biomarkers, which thereby certifies the significance and necessity of the present study.

Pyroptosis, another form of programmed cell death, is also called inflammatory necrosis of cells and has the features of constant cell expansion till the rupture of cell membrane; as a result, cell contents are released and a strong inflammatory response is induced [14]. Many studies suggest that healthy tissue can be transformed in cancerous one in the inflammatory environment, and the pyroptosis-mediated inflammatory response environment helps cancer development and accelerates cancer cell apoptosis [15]. Caspase-1 protein can be recruited and activated in the body via the canonical inflammasome pathways; meanwhile, caspase 4/5/11 proteins can also be activated via noncanonical inflammasome pathways for the direct cleavage and activation of gasdermin protein D (GSDMD), finally causing membrane pore formation as well as cell death. Pyroptosis has an important effect on tumor genesis and development [16]. Different pyroptosis-associated factors, like genes in gasdermin family, inflammatory vesicles, and proinflammatory factors, are tightly related to cancer genesis and metastasis [17]. Besides, pyroptosis has a critical anticancer effect through the activation of immune response. Through the pores on cell membrane, cells experiencing pyroptosis can dissociate plenty of intracellular contents, thus inducing massive lymphocyte infiltration and potent inflammatory response. Due to the dramatically enhanced lymphocyte infiltration, the pyroptosis of cancer cells mediated by the caspase-3-independent and caspase-3-dependent pathways is aggravated, which leads to the formation of positive feedback for enhancing the anticancer activity [18]. Cao et al. constructed a signature by incorporating the pyroptosis-associated genes to predict SKCM survival [19]. However, the associations between pyroptosis characteristics and TIME features as well as treatment response remain to be further explored among individual cases, so as to help to diagnose patients and develop personal treatment.

This study comprehensively evaluated the roles and prognostic value of pyroptosis-associated genes (PAGs) within the SKCM TIME. We downloaded mRNA expression and clinical data of SKCM patients in GEO and TCGA datasets. In addition, we conducted ssGSEA for evaluating pyroptosis-associated profiles in patients and exploring the relations with clinical characteristics and patient survival. Further, weighted gene coexpression network analysis (WGCNA) was performed in identifying the key genes/modules related to pyroptosis-associated profiles. Thereafter, we built a risk signature based on PAGs (called PPRS for short) on the basis of TCGA cohort and verified PPRS using additional 3 GEO datasets. Further, we also constructed a nomogram integrating PPRS and clinical features for assessing SKCM survival. Findings in this work also help to illustrate the tumor stemness, immune landscape, and treatment response among SKCM patients by different PPRS. Collectively, we combined genomic data for the thorough assessment of relation of pyroptosis with infiltration features of tumor immune microenvironment (TIME) within SKCM, as well as the possible clinical value and molecular roles of PAGs; besides, this study also provides the putative prognostic biomarkers for melanoma.

## 2. Materials and Methods

### 2.1. SKCM Datasets and Pyroptosis-Regulated Genes

We obtained the RNA-seq profiling data (FPKM values) for SKCM patients in TCGA database [20] as the training set. Afterwards, samples with incomplete clinical data were eliminated, leaving 363 melanoma patients being enrolled into the present work. Thereafter, we transformed FPKM values into TPM values by TPM*i* = FPKM*i* × 1000000/(FPKM0+⋯.+FPKM*m*). In the formula, *i* stands for gene *i*, whereas *m* is indicative of overall gene number. In addition, we obtained CNVs and somatic mutations in TCGA database. Further, we also downloaded expression and clinical information in GEO datasets as the test sets, which included GSE22153 (*n* = 54) [21], GSE54467 (*n* = 79) [22], and GSE65904 (*n* = 188 [23] after eliminating samples that had incomplete prognostic information). Moreover, we searched the MSigDB database [24] (keywords: REACTOME_PYROPTOSIS) to identify pyroptosis-associated markers, suppressive, and driver genes. After removing the duplicates, we obtained 27 candidate pyroptosis-regulated genes in later analyses.

### 2.2. ssGSEA

For constructing pyroptosis-associated profiles among SKCM cases, ssGSEA was carried out by “GSVA” in R package. Gene set variation analysis (GSVA) [25] has been developed as the unsupervised, nonparametric approach for determining gene set enrichment variations based on pyroptosis-regulated gene expression dataset samples. The ssGSEA enrichment scores stand for the upregulation or downregulation degrees of genes in the sample. Those critical parameters were shown as follows: min.sz = 1, kcdf = “Gaussian,” abs.ranking = TRUE, tau = 0.25, and max.sz = Inf. We acquired gene sets in MSigDB databases.

### 2.3. Calculation of the Stemness Index (mRNAsi)

Based on one-class logistic regression (OCLR) algorithm, the stemness index model trained from the Progenitor Cell Biology Consortium database [26] was used to calculate tumor stemness. The stemness index can be used to measure how similar tumor cells are to stem cells, with stemness index being a value between 0 (lowest) and 1 (highest). The closer the stemness index is to 1, the stronger the stem cell properties.

### 2.4. Immune Pathway Activities, Immune Infiltration Levels, and Immunotherapeutic Response

This work utilized ESTIMATE algorithm in calculating tumor purity and ESTIMATE/stromal/immune scores for every melanoma sample. Besides, this study adopted CIBERSORT [27] in estimating infiltration degrees of diverse immune cells within TIME. Additionally, we assessed immunotherapeutic response by analyzing immunophenoscore (IPS) [28]. According to prior description, the IPS of one case may be obtained with no bias by machine learning after considering 4 main immunogenicity-determining gene classes, including immunosuppressive cells, effector cells, immunomodulators, and MHC molecules. It can be completed through gene expression profile analysis within cell types of those 4 classes. IPS can be determined by the scale ranging from 0 to 10 according to gene expression *Z*-scores in typical cell types, and a high score stands for the higher immunogenicity. We obtained IPSs of SKCM cases in The Cancer Immunome Atlas (TCIA, https://tcia.at/).

### 2.5. Weighted Gene Coexpression Network Analysis (WGCNA)

This work adopted WGCNA [29] for identifying key coexpression modules related to pyroptosis-associated profiles and analyzing gene transcription profiles. Meanwhile, we extracted gene expression profiles from TCGA, and the cluster threshold, *β*-value, and median absolute deviation were 5, 7, and > 50%, separately. Thereafter, we converted the expression matrix into topology matrix and obtained modules by mean linkages using parameters as follows: height = 0.3, deep split = 2, and min module size = 30.

### 2.6. Establishment and Estimation of PPRS for Prognosis Prediction of SKCM

Firstly, PAGs markedly linked with eigengenes in coexpression modules related to pyroptosis-associated profiles were screened by *P* < 0.01 threshold. Subsequently, univariate Cox regression was performed to search prognostic PAGs. Additionally, LASSO Cox regression was conducted to establish the PPRS with the least risk of overfitting. LASSO algorithm was utilized to select variables using glmnet in R package. Moreover, PPRS of each case was determined according to gene levels and related regression coefficients. *Z*-score was used to normalize the values of PPRS, and SKCM patients from training and test sets were classified into high-risk (*Z*‐score ≥ 0) and low-risk (*Z*‐score < 0) groups. Afterwards, survival and survminer in R package were utilized for plotting survival curves, while log-rank test was conducted for comparing 2 groups. Later, values of area under the time-dependent receiver operating characteristic (t-ROC) curves (AUC) were determined for predicting OS at 1, 3, and 5 years. We also adopted Wilcoxon's rank sum test for analyzing the associations between clinical characteristics, oncogenic mutations, ICI contents, immune infiltration degrees, biological processes (BPs), and treatment response with PPRS.

### 2.7. Construction and Confirmation of the Prediction Model

This study constructed the nomogram based on the previously discovered eligible factors via rms, survival, and foreign in R package. Moreover, ROC curves together with calibration curves were plotted to evaluate the discriminating ability and calibration of the as-constructed nomogram.

### 2.8. Statistical Analysis

We applied Fisher's exact test and chi-square test for analyzing the associations of clinical variables with diverse SKCM types, and the two-tailed *P* < 0.05 was considered as statistically significant. The Wilcoxon tests were used to compare variables with normal distribution between 2 groups, whereas those with abnormal distribution were compared through unpaired Student's *t*-tests. One-way ANOVA was the parametric approach, whereas the Kruskal-Wallis test was the nonparametric approach, and they were adopted for comparing differences among several groups. The Pearson and distance correlation analyses were utilized to determine correlation coefficients. We also applied the two-sided Fisher's exact test to analyze contingency tables. Meanwhile, the Kaplan-Meier (KM) approach was utilized to draw cluster survival curves, while log-rank test was applied in statistical difference analysis. Furthermore, we also performed FDR correction with multiple testing for reducing false-positive rate (FPR). All statistical analyses were completed with R software (version 3.5.3, http://www.R-project.org).

## 3. Results

### 3.1. Landscape Showing the Genetic Variations for 27 Pyroptosis-Regulated Genes within SKCM

**Figure 1:**
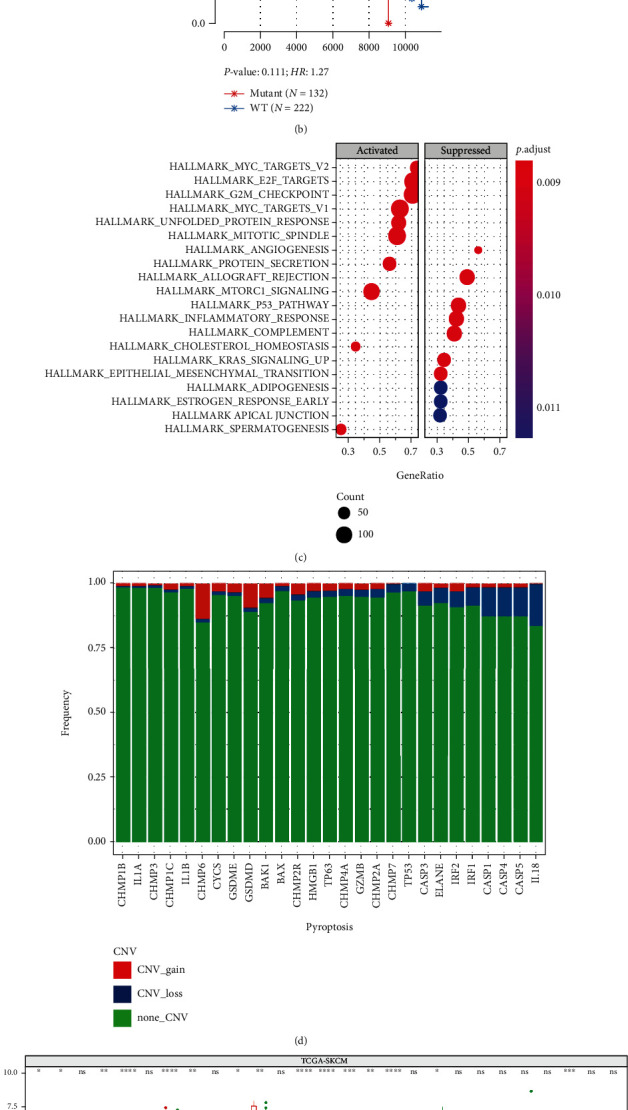
Landscape showing the genetic variations for 27 pyroptosis-regulated genes within SKCM. (a) Mutation frequency of pyroptosis-regulated genes in TCGA-SKCM samples. (b) Difference in prognosis between patients with the pyroptosis-regulated gene mutations and wild-type (WT) patients. (c) Enriched pathways in pyroptosis-regulated gene mutations and WT patients. (d) CNV frequency of pyroptosis-regulated genes in TCGA-SKCM samples. (e) Pyroptosis-regulated genes harboring CNV amplification displayed high expression within SKCM. (f) The differential expression in pyroptosis-regulated genes between metastatic and primary tumor patients.

We obtained 27 pyroptosis-regulated genes from MSigDB database. Of all the 363 SKCM-TCGA samples, we found mutations in 134 samples, and the mutation frequency was 36.9% (Figure 1(a)). A majority of samples harbored the nonsense mutations. Typically, TP63 and TP53 had comparatively higher mutation frequency (more than 40%), while no mutation in CHMP2A was detected in TCGA-SKCM samples.

Subsequently, after excluding samples without survival data, we compared the difference in prognosis between patients with the mutations and wild type (WT) of the above-mentioned genes. The results suggested that the overall survival (OS) between two groups was not significant (Figure 1(b)).

The subsequent GSVA results revealed that MUT samples were mainly enriched into the cell cycle-related pathways, such as Myc and E2F, while the immune-related pathways were enriched in WT samples (Figure 1(c)). This revealed that the pyroptosis-regulated genes might affect SKCM progression through affecting the cell cycle of tumor cells and immune response in TIME. By analyzing CNV frequency, 27 pyroptosis-regulated genes showed infrequent CNVs, with a majority of them being copy number amplification, whereas CHMP6, GSDMD, and BAK1 exhibited the general CNV-gain frequency, while IL-18 and CASP1,4,5 displayed the wide CNV-loss frequency (Figure 1(d)). Further, pyroptosis-regulated genes harboring CNV amplification displayed high expression within SKCM (Figure 1(e)). Finally, we compared the differential expression in pyroptosis-regulated genes between metastatic and primary tumor patients. The results suggested that most genes were significantly different between two groups, among which, CASP3, CASP5, CHMP2B, CHMP7, GZMB, IRF1, and IRF2 were highly expressed in metastatic patients, whereas BAX, CHMP2A, CHMP4A, CHMP4B, CHMP4C, IL1A, and TP63 were highly expressed in primary tumors (Figure 1(f)). These analyses suggested the highly heterogenous pyroptosis-regulated gene expression and genetic profiles within SKCM, which indicated the probable function of pyroptosis during cancer genesis and progression.

### 3.2. Establishment of Pyroptosis-Associated Profiles among SKCM Cases as well as the Relation with Additional Clinical Variables and Prognostic Outcome

**Figure 2:**
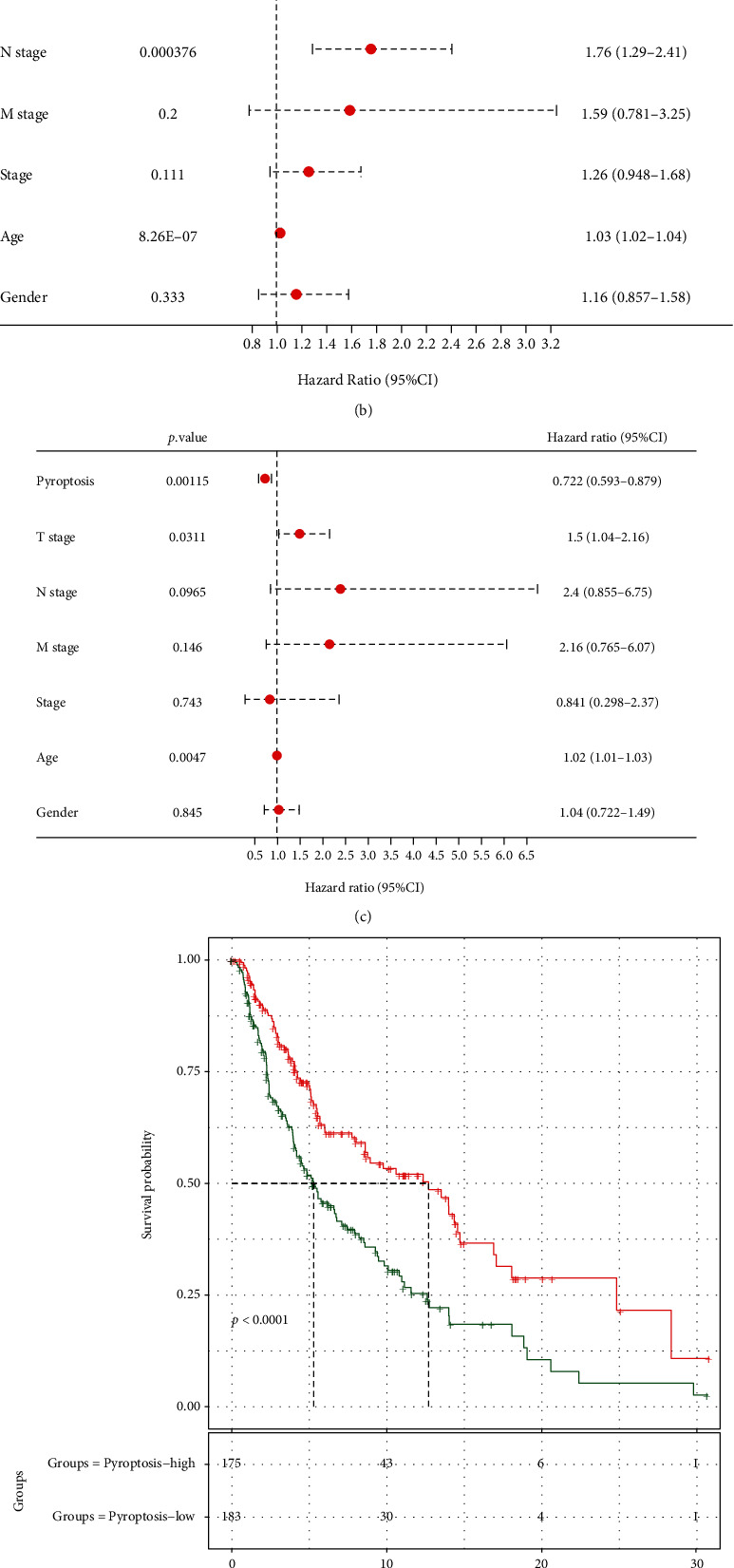
Establishment of pyroptosis-associated profiles among SKCM cases as well as the relation with additional clinical variables and prognostic outcome. (a) Correlation analysis between the scores of pyroptosis-associated profiles and clinicopathological features of melanoma patients in TCGA-SKCM cohort. (b, c) Univariate (b) and multivariate (c) Cox regressions were used to analyze the independent prognostic factors for melanoma. (d) KM analysis between samples with high and low scores of pyroptosis-associated profiles. (e) The difference in scores of pyroptosis-associated profiles between different clinicopathological feature groups.

Based on the 27 pyroptosis-regulated genes, we adopted ssGSEA to quantify the scores of pyroptosis-associated profiles of each sample in the TCGA-SKCM training set. After *Z*-score normalization, the samples were divided into pyroptosis-low (*Z*‐score < 0) and pyroptosis-high (*Z*‐score ≥ 0) groups. Then, we conducted correlation analysis between the scores of pyroptosis-associated profiles and clinicopathological features of melanoma patients, which suggested that the scores of pyroptosis-associated profiles were significantly correlated with T stage (negative) and patient survival (positive) (Figure 2(a)). Further, we performed univariate and multivariate COX analyses, which indicated that scores of pyroptosis-associated profiles were the protective factor that affected the OS of SKCM patients, whereas T stage and N stage were the major risk factors that affecting OS (Figures 2(b) and 2(c)). Next, based on the scores of pyroptosis-associated profiles, we divided melanoma samples from TCGA-SKCM dataset into high- and low-score groups. Survival analysis suggested that the OS rate in the high-score group was higher (Figure 2(d)). Moreover, we also compared the difference in scores of pyroptosis-associated profiles between different clinicopathological feature groups (Figure 2(e)). As a result, the scores of pyroptosis-associated profiles decreased with the increase in clinical stage. On the whole, patients with high scores of pyroptosis-associated profiles had high OS rate, and pyroptosis was the main protective factor for the prognosis of melanoma patients.

### 3.3. Key Gene Modules Linked with Pyroptosis-Associated Profiles Detected by WGCNA

**Figure 3:**
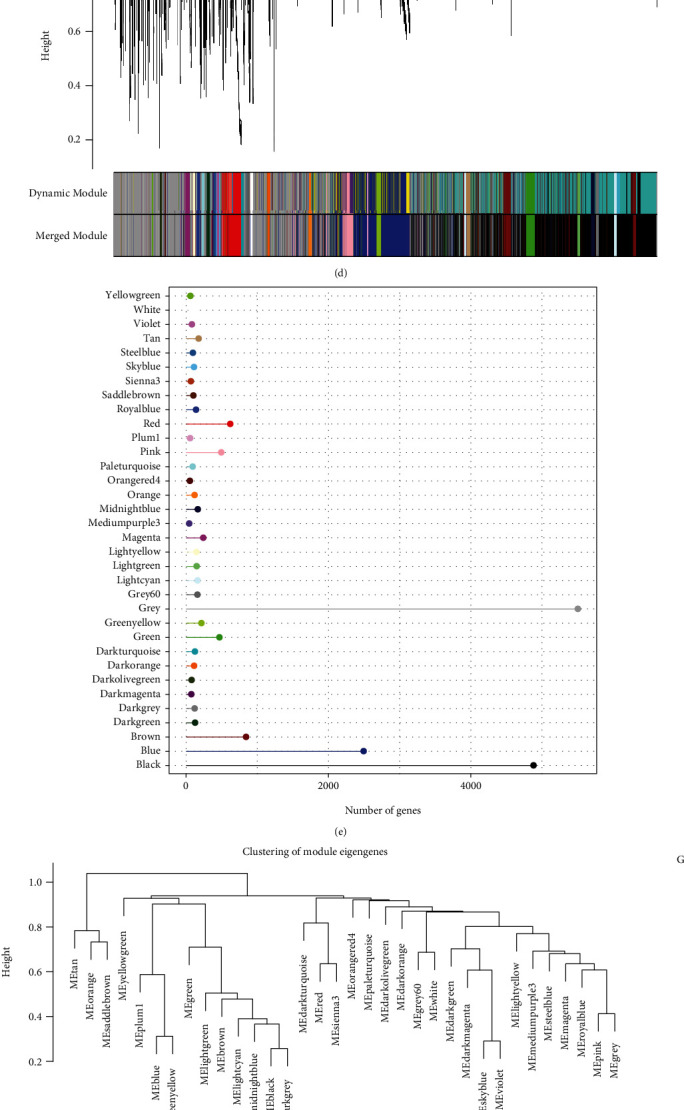
WGCNA on genes related to the pyroptosis-associated profiles among melanoma cases in TCGA-SKCM cohorts. (a) Clustering tree of each sample. (b) Analysis of the scale-free fit index for various soft-thresholding powers (*β*). (c) Analysis of the mean connectivity for various soft-thresholding powers. (d) Dendrogram of all differentially expressed genes clustered based on a dissimilarity measure (1-TOM). (e) Gene numbers within those 34 coexpression gene modules. (f) Heat map presenting the relations of modules with scores of pyroptosis-associated profiles. (g) Scatter diagram for module membership vs. gene significance in the blue module.

Firstly, we classified SKCM cases based on distributed-cluster analysis according to the gene expression profiles (Figure 3(a)). To guarantee the scale-free network, this study sets the soft thresholding at *β* = 7 (Figures 3(b) and 3(c)). Afterwards, the representation matrix was converted into the adjacency one and subsequently into the topological one. Thereafter, we classified genes by average-linkage hierarchy clustering. According to the hybrid dynamic shear tree standard, there were at least 30 genes in each gene network module. Dynamic shearing was then conducted to determine gene modules, followed by the calculation of eigengene values. Later, we carried out clustering analysis in each module and combined neighbour modules into the combined one using the following parameters: deep split = 2, height = 0.3, and min module size = 30. This study obtained 34 modules in total (Figures 3(d) and 3(e)). Additionally, we analyzed the relations of eigenvectors for the 34 modules with pyroptosis-associated profile scores. Specifically, blue module was apparently positively related to pyroptosis-associated profiles among SKCM cases (Figure 3(f), *r* = 0.84, *P* < 1*e*^−5^). Likewise, in blue module, gene significance (GS) was positively related to module membership (MM) (Figure 3(g), *r* = 0.98, *P* < 1*e*^−5^). Finally, we verified that the blue module was the key gene module of pyroptosis in SKCM.

### 3.4. PPRS Construction and Validation

**Figure 4:**
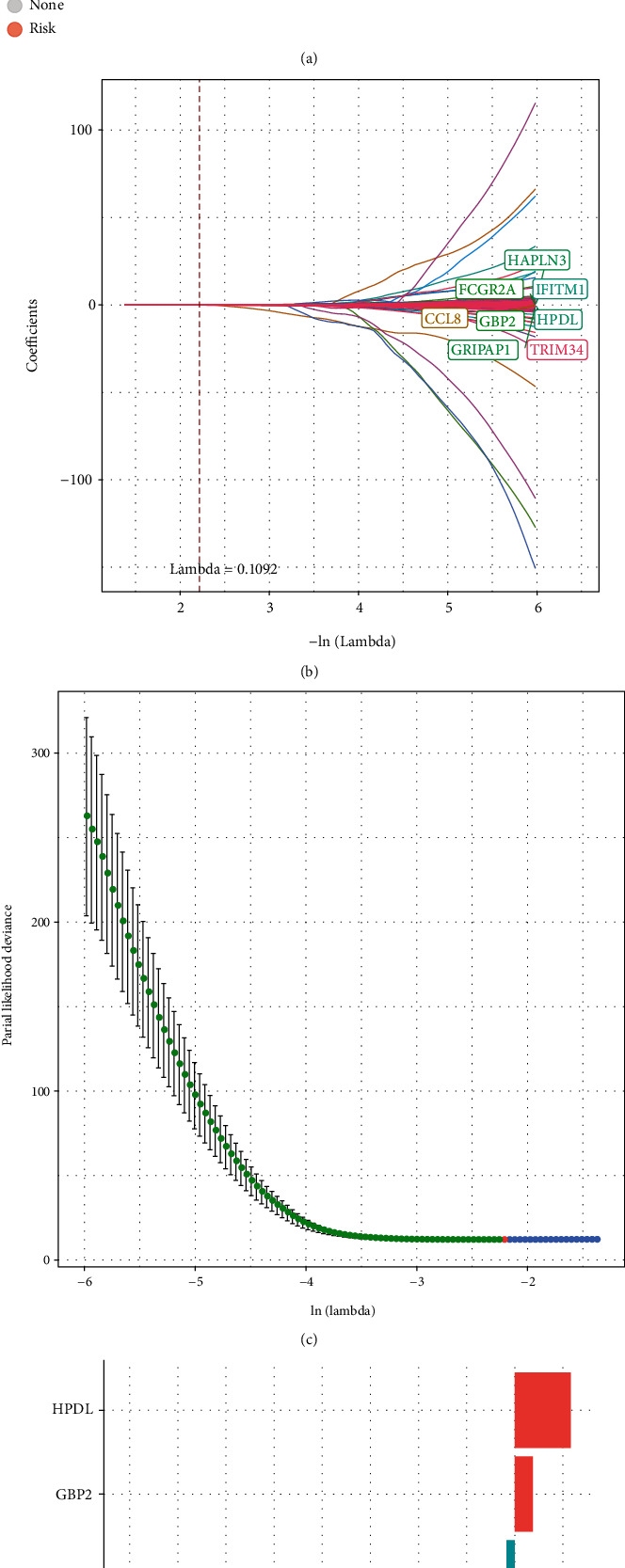
Construction of the PPRS for melanoma patients. (a) 984 prognostic genes were obtained by univariate Cox regression analyses. (b) The changing trajectory of each independent variable. The horizontal axis represents the log value of the independent variable lambda, and the vertical axis represents the coefficient of the independent variable. (c) Confidence intervals for each lambda. (d) Distribution of LASSO coefficients of PPRS.

First of all, we identified genes significantly positively related to eigenvectors in the blue module (*P* < 0.01), which were the hub PAGs. Later, to explore whether the selected genes were significant, univariate Cox regression was carried out on the basis of clinical prognostic data. Results of *P* < 0.01 were recorded, and altogether, 984 prognostic genes were obtained (Figure 4(a), 900 prognosis protective and 84 risk genes). Then, results obtained from univariate Cox regression (*P* < 0.01) were integrated into LASSO regression. The dimension was reduced, and lambda as well as proportional hazards model curves revealed that when there were 8 genes in the model, the smallest deviance was achieved (Figures 4(b)–4(d), lambda = 0.1092, GBP2, HPDL, FCGR2A, IFITM1, HAPLN3, CCL8, TRIM34, and GRIPAP1). PPRS was calculated by the following formula:

(1) $$\begin{array}{c} \text{PPRS}\left(\text{RiskScore}\right)=-0.166\times \text{CCL}8-0.156\times \text{FCGR}2\text{A}+0.047\times \text{GBP}2-0.327\times \text{GRIPAP}1-0.207\times \text{HAPLN}3+0.145\times \text{HPDL}-0.022\times \text{IFITM}1-1.01\times \text{TRIM}34. \end{array}$$

**Figure 5:**
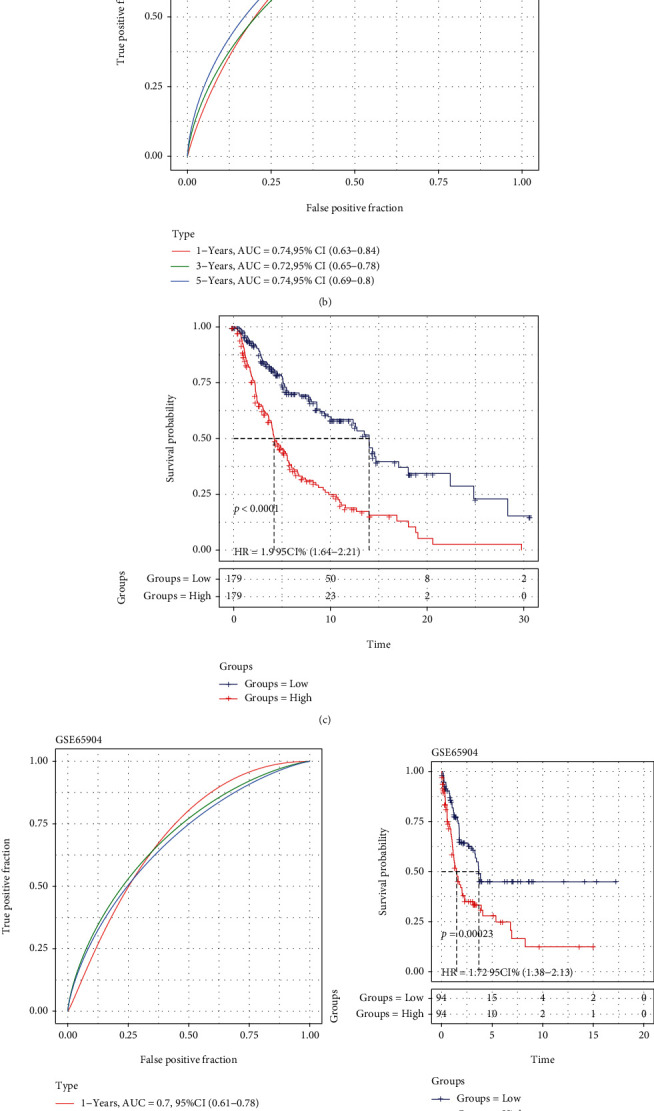
Association of patients' survival with PPRS from both training and test cohorts. (a) 8 PAG expression levels, PPRS score, status, and survival time in each case from TCGA-SKCM dataset. (b) Performance of PPRS in predicting prognosis analyzed by 1-, 3-, and 5-year ROC curves in TCGA-SKCM dataset. (c) KM analysis between high and low PPRS score patients from TCGA-SKCM dataset. (d) Performance of PPRS in predicting prognosis in GSE65904 dataset. (e) Performance of PPRS in predicting prognosis in GSE54467 dataset. (f) Performance of PPRS in predicting prognosis in GSE22153 dataset.

Figure 5(a)exhibits expression profiles for 8 prognostic PAGs, survival status, and PPRS distribution of training set samples. According to our results, samples that had increased RiskScore were associated with the markedly decreased OS relative to samples having low RiskScore. Further, the upregulation of GBP2 and HPDL predicted an increased risk, which were the risk factors. However, for another 6 PAGs, their upregulation predicted the decreased risk, which were identified as protective factors. In this study, TimeROC in R software was adopted to classify the prognosis by using PPRS (Figure 5(b)). As a result, the constructed PPRS had great AUC values for 1-, 3-, and 5-year OS (0.74, 0.72, and 0.74, separately). Eventually, this study analyzed PPRS based on *Z*-score, so as to classify cases as the high-score (scores > 0, *n* = 179) and low-score (scores < 0, *n* = 179) groups. According to KM curve analysis (Figure 5(c)), there was significant difference in survival between 2 groups (*P* < 0.0001, HR = 1.9, 95% CI: 1.64-2.21).

For evaluating whether our constructed PPRS was effective on prognosis prediction, we utilized 3 external test sets for validating (Figures 5(d)–5(f)). We obtained AUC values of 0.66-0.95 in the prediction of 1-, 3-, and 5-year OS in those 3 test sets. This study also divided, respectively, 94 and 94 samples from GSE65904 cohort (Figure 5(d)), 39 and 40 from GSE54467 cohort (Figure 5(e)), and 27 and 27 samples from GSE22153 (Figure 5(f)) into low- and high-score groups, with the survival difference being significant between 2 groups (GSE65904: *P* = 0.00023, HR = 1.72, 95% CI: 1.38-2.13; GSE54467: *P* = 0.0079, HR = 1.96, 95% CI: 1.41-2.72; GSE22153: *P* = 0.0022, HR = 2.93, 95% CI: 1.95-4.4).

In order to further verify the efficiency of PPRS in predicting the prognosis of melanoma, we selected three reported prognosis prediction models, including 4-gene signature [30], 12-gene signature [31], and 5-gene signature [32], to compare with our constructed PPRS. The gene expression data of TCGA-SKCM samples were utilized to calculate the RiskScore of each sample in each prognosis prediction model. Subsequently, the RiskScore was transformed into *Z*-score, and samples with the value of ≥0 were classified into high-risk group, while those of <0 were classified to low-risk group. Then, the difference in OS between two groups of samples was calculated. The ROC and KM curves of three models are shown in Figure S1 A-C. It was seen that all of the three models had lower ROC values to PPRS, and the difference in OS between high- and low-risk groups was statistically significant. In addition, we used the C-index (concordance index) to evaluate the predictive ability of the 4 models. As shown in Figure S1 D, the C-index of PPRS was higher than that of the other three risk models. To sum up, our model was a relatively reasonable, effective, and clinically convenient prognosis prediction model for SKCM with relative fewer genes.

### 3.5. Relations of Clinical Characteristics and Pathways with PPRS

**Figure 6:**
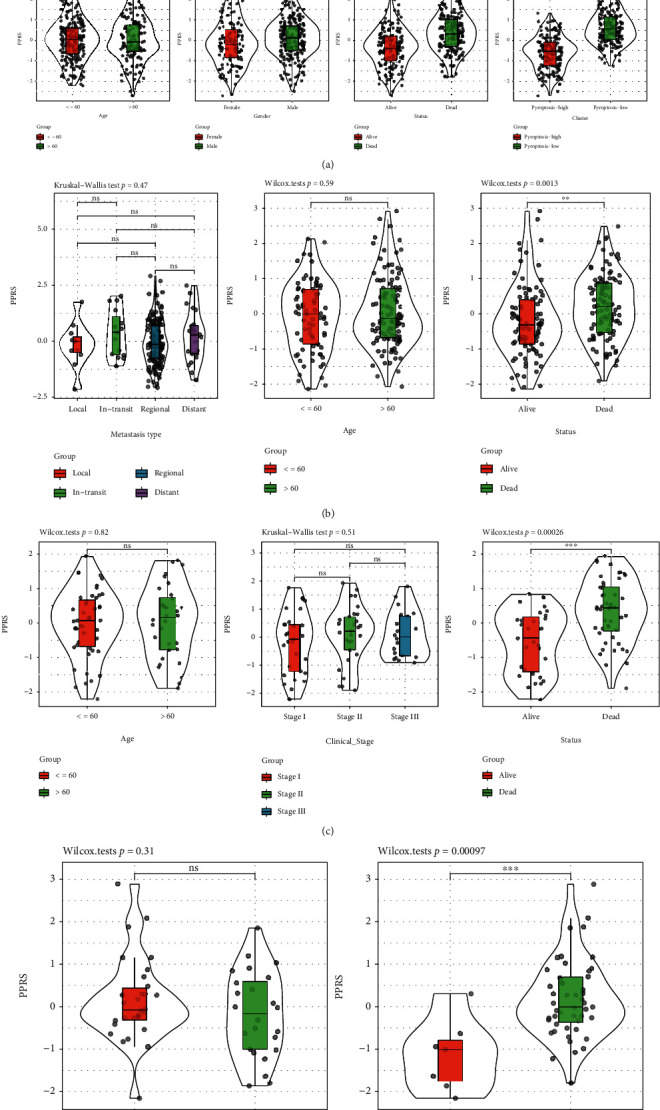
The distribution of PPRS levels in samples with different clinicopathological features from TCGA-SKCM (a), GSE65904 (b), GSE54467 (c), and GSE22153 (d) cohorts.

First, this study compared PPRS distribution among diverse clinicopathological characteristics groups. As shown in Figures 6(a)–6(d), PPRS markedly raised as death status and T stage elevated, both for training and test set samples. At the same time, the difference in PPRS was not significant among diverse ages.

**Figure 7:**
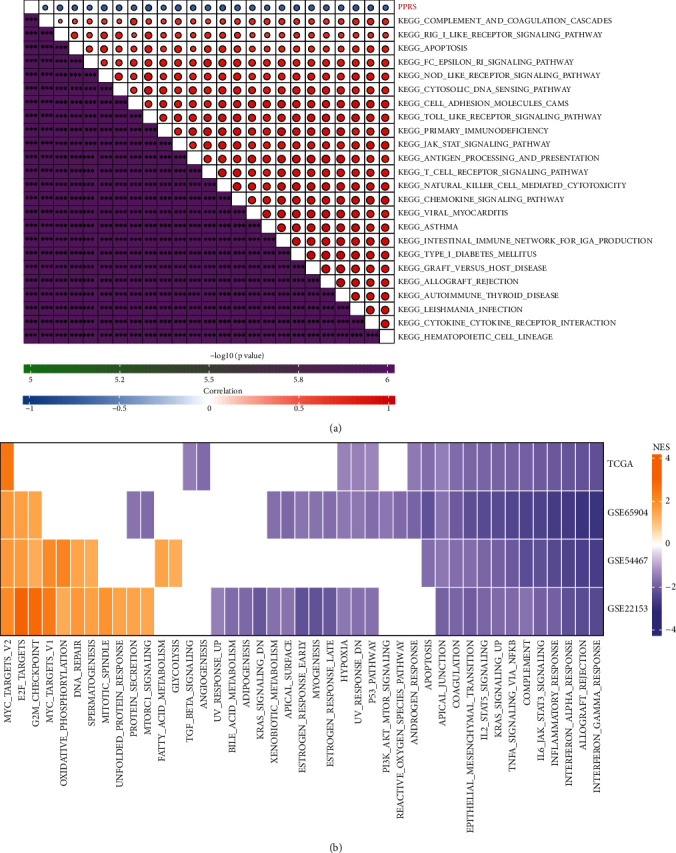
The relation between pathways and PPRS. (a) Correlation analysis between PPRS and KEGG pathways through ssGSEA. (b) A heat map demonstrating normalized enrichment scores (NESs) of pathways in MSigDB calculated by comparing PPRS-high with PPRS-low groups (with a false discovery rate (FDR) of <0.05).

Then, ssGSEA was performed among pathways enriched by TCGA-SKCM samples through GSVA, so as to analyze the association of pathways with PPRS. As a result (Figure 7(a)), most pathways showed negative relation to the PPRS of samples (with the correlation coefficients > 0.6). Meanwhile, we analyzed the differences in pathway activation/suppression in samples showing diverse PPRS values. As a result (Figure 7(b)), relative to low PPRS samples from TCGA-SKCM cohort, 1 pathway was activated among the high PPRS samples, whereas 19 were inhibited. In addition, there were 3, 9, and 11 pathways being activated among high PPRS samples from GSE65904, GSE54467, and GSE22153 cohorts. Overall, cell cycle activation along with immunoinflammatory response pathway suppression was the possible risk factor for samples with higher PPRS scores.

### 3.6. Relations of Tumor Stemness with PPRS

**Figure 8:**
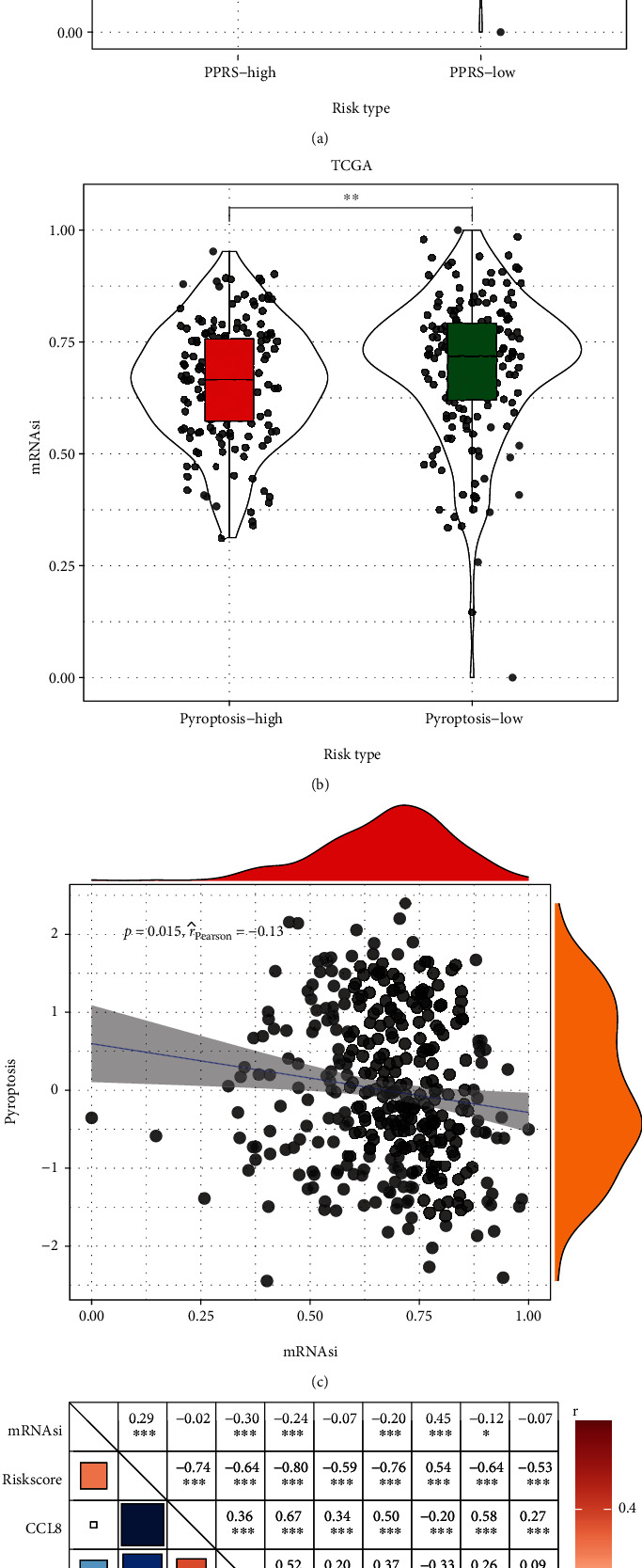
Relation between ability of tumor stemness and PPRS. (a) Differences in levels of mRNAsi between samples with diver risk scores. (b) Differences in levels of mRNAsi between samples in different pyroptosis statuses. (c) Correlation between mRNAsi and pyroptosis statuses. (d) Correlation between the levels of risk scores, GBP2, HPDL, FCGR2A, IFITM1, HAPLN3, CCL8, TRIM34, GRIPAP1, and tumor stemness.

Based on the critical role of cancer stem cell-like cells (CSCs) on deregulation of cell cycle resulting in the abnormal proliferation and differentiation of tumor, we explored the association between tumor stemness and our constructed PPRS. We scored the stemness index for each sample in the TCGA-SKCM cohort and examined differences in the levels of mRNAsi between samples with different PPRS or pyroptosis statuses (scores of pyroptosis-associated profiles). As shown in Figures 8(a) and 8(b), samples in the high-risk or pyroptosis-low group with poor prognosis had higher stem cell properties. The pyroptosis status of the samples showed a significant negative correlation with mRNAsi (Figure 8(c)). At last, the key PAG that constitute the risk model, HPDL, was found to be significantly positively correlated with tumor stemness (Figure 8(d)). The above results partially confirmed that tumor stemness is closely related to the pyroptosis Statuses and prognosis of SKCM patients, while the regulatory roles of the HPDL in stem cell properties need to be further explored.

### 3.7. Relation between Immune Infiltration, Immunotherapeutic and Chemotherapeutic Responses, and PPRS

**Figure 9:**
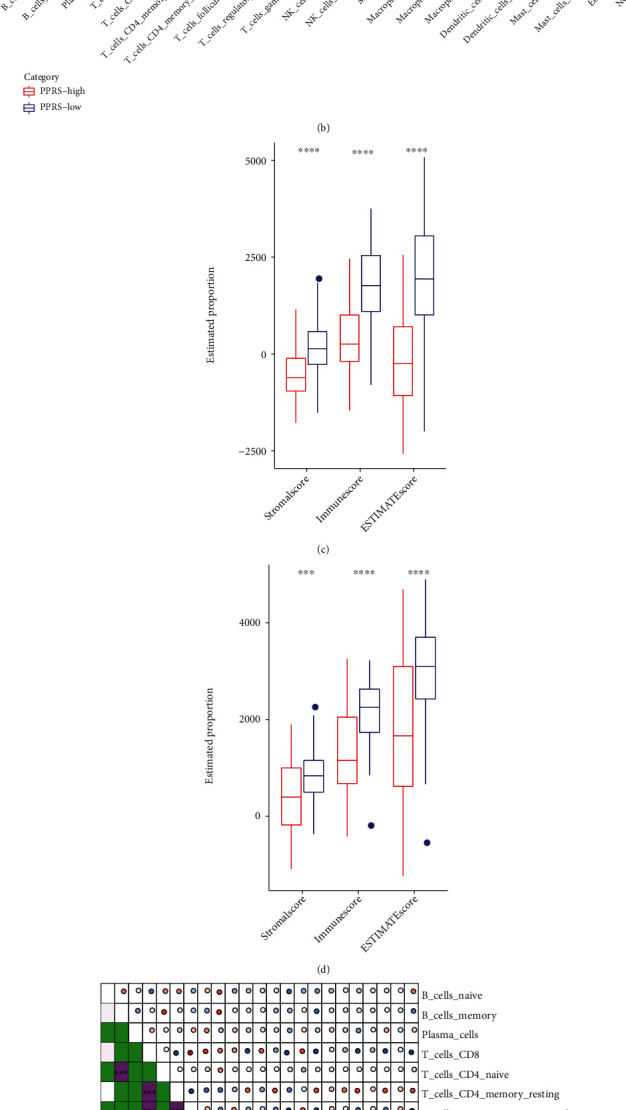
Immune profiles of melanoma samples with distinct PPRS score. (a, b) Immune infiltration degrees within TIME for melanoma samples from TCGA-SKCM (a) and GSE65904 (b) datasets with high and low PPRS scores by CIBERSORT approach. (c, d) ESTIMATE/immune/stromal scores for melanoma samples from TCGA-SKCM (c) and GSE65904 (d) datasets with high and low PPRS scores. (e) The relation between immune infiltrations and PPRS.

This article examined the association of PPRS level with TIME. Firstly, we examined the relation between immune infiltrates and PPRS. As a result (Figures 9(a) and 9(b)), PPRS exhibited obvious positive and negative relations to M2 and M1 macrophages, respectively. Moreover, differences in T lymphocytes were of statistical significance between 2 groups (*P* < 0.001). Secondly, to determine the relation between stromal/immune cell percentages and PPRS, this work also determined ESTIMATE/immune/stromal scores for patients with high and low PPRS scores using ESTIMATE in R package. As shown in Figures 9(c) and 9(d), there were significant differences in immune, stromal, and ESTIMATE scores between samples with diverse PPRS scores from both TCGA and GSE65904 cohorts. Taken together (Figure 9(e)), our immune infiltration analysis demonstrated the distinct differences in TIME features between low and high PPRS patients, and it facilitates to develop immunotherapy against SKCM.

**Figure 10:**
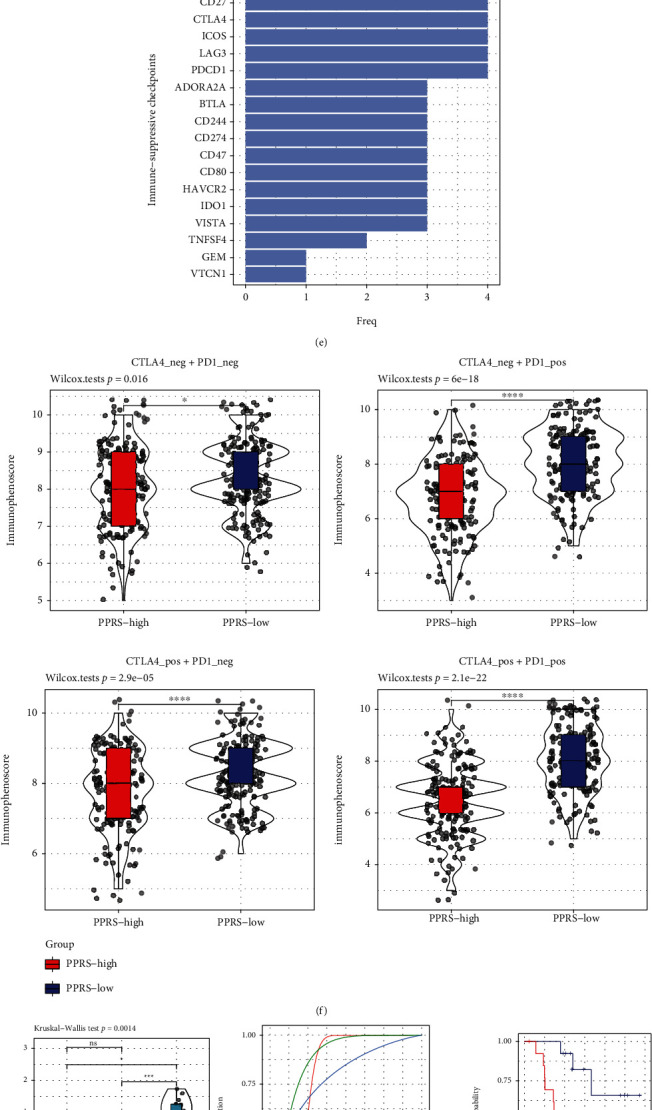
Diverse immunotherapeutic responses among melanoma samples from PPRS-high and PPRS-low groups. (a–d) IC levels within melanoma samples from TCGA-SKCM (a), GSE65904 (b), GSE54467 (c), and GSE22153 (d) cohorts with high and low PPRS scores. (e) A bar plot demonstrating frequencies of immune checkpoints upregulated in PPRS-low cancer patients across the four cohorts. The *y*-axis indicates the names of immune checkpoints, and the *x*-axis represents the number of cohort. (f) The boxplots indicate the average immunophenoscore values IPS across the two PPRS subgroups in SKCM tumors. Overall, PPRS-low tumors that could be treated with combined anti-PD-1 and anti-CTLA-4 checkpoint blockade or with anti-PD-1 alone had significantly higher IPS, which is indicative of a better response to these immunotherapies. (g, h) The distribution of PPRS levels in samples responsive and resistant to treatment from GSE78220 (g) and GSE91061 (h) datasets and its performance in predicting samples prognosis. (i) Box plots exhibiting the estimated IC_50_ values of temozolomide, paclitaxel, and cisplatin within melanoma samples from TCGA-SKCM dataset with high and low PPRS scores.

Further, ICIs may be utilized in immunotherapy. Therefore, the present work analyzed immune checkpoint expression in samples from both training and test sets. We found that levels of these genes were significantly different between low and high PPRS groups, among which, most were highly expressed in patients with high PPRS (Figures 10(a)–10(e)). Such results suggested the significant differences in treatment response between 2 groups, in particular for immunotherapy.

For verifying this hypothesis, IPS analysis was conducted for comparing the different immunotherapeutic responses in diverse PPRS samples. The present work determined IPS scores for predicting whether ICIs might be adopted among cases. As the result, these scores dramatically elevated among patients with low PPRS (Figure 10(f)). Based on the above results, patients with low PPRS scores were associated with the increased IPS and a higher probability of immunogenic phenotype, making them the candidates for ICIs. Furthermore, 2 datasets that contained complete gene expression profiles, treatment response to PD-1 monoclonal antibody, and clinical data for melanoma were obtained from GSE78220 [33] and GSE91061 [34] datasets. PPRS levels for treatment-resistant or responsive patients were determined. As a result (Figures 10(g) and 10(h)), PPRS of responsive patients markedly declined relative to resistant (SD and PD) patients. At last, samples from GSE78220 and GSE91061 datasets were classified as the low or high PPRS score group to compare the prognosis. Based on our results (Figures 10(g) and 10(h)), PPRS was able to predict prognostic outcome and classify patient survivals at 6 months, 1 year, and 2 years. Great AUC values were obtained, and the difference in survival was significant between high and low PPRS groups.

At last, this study examined responses to traditional chemotherapeutics among samples from TCGA cohort of diverse PPRS groups. According to our results (Figure 10(i)), patients with low PPRS showed higher sensitivity to temozolomide and paclitaxel, but those with high PPRS exhibited higher sensitivity to cisplatin.

### 3.8. Clinical Model Construction to Precisely Stratify Risk in SKCM Patients

**Figure 11:**
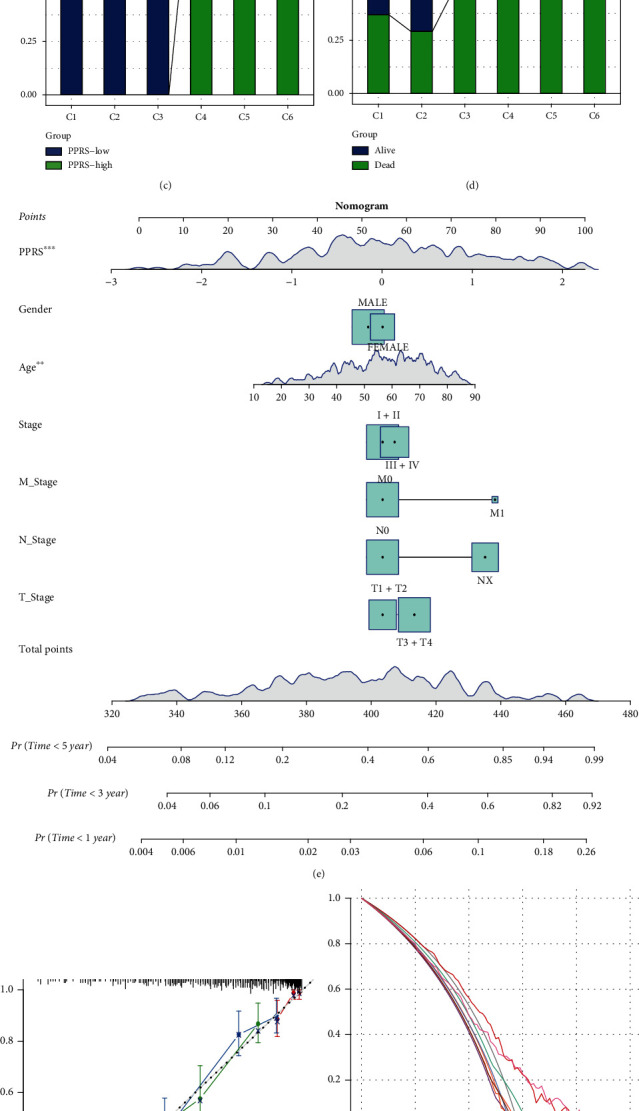
The nomogram and survival decision tree were produced for improving risk stratification and predicting the survival probability. (a) Cases who had complete annotations such as T stage, N stage, PPRS, and age were applied in constructing the survival decision tree for optimizing risk stratification. (b–d) Differences in OS (b), PPRS scores (c), and living states (d) were significant across the 6 risk groups. (e) Detailed information of the nomogram. (f) Our constructed PPRS and nomogram were highly accurate based on calibration analysis. (g) Decision-making curve of the nomogram. (h) Relative to additional clinicopathological factors, our as-constructed PPRS and nomogram performed well in predicting survival.

In total, 358 cases from TCGA cohort that had sufficient clinical information such as gender, age, histology, TNM stage, PPRS, and grade were selected for recursive partitioning analysis to construct the adjustable model. Age, PPRS, M stage, and N stage were selected to construct the eventual decision tree model; according to their distribution levels, 6 risk subgroups were obtained (Figure 11(a)). The difference in patient survival was significant across those 6 risk subgroups (Figure 11(b)). Typically, cluster_1 samples showed the most favorable prognostic outcome, while cluster_6 samples exhibited the worst survival, had high PPRS, and were distributed in higher N stage. Meanwhile, survival status and PPRS distributions were compared across those 6 risk subgroups. As a result, compared with cluster_1 samples, cluster_6 samples had an increased rate of “dead” survival status (Figures 11(c) and 11(d)).

Besides, we selected those significant variables in constructing a nomogram (Figure 11(e)). As revealed by calibration analysis, the survival curves at 1, 3, and 5 years approached the optimal 45-degree calibration line, indicating that the as-constructed nomogram was accurate (Figure 11(f)). Based on decision curve analysis (DCA), nomogram and PPRS achieved greater survival net benefits than additional factors (Figure 11(g)). Additionally, 5-year ROC curve revealed that nomogram and PPRS attained the highest accuracy in predicting survival compared with the remaining clinical variables (Figure 10(h)).

## 4. Discussion

TNM classification system [35] has been widely adopted for assessing SKCM survival. However, it can not accurately predict prognosis due to the related practical limitations. In this regard, it is of great importance to discover the appropriate biomarkers to judge tumor grade, evaluate patient prognosis, select appropriate treatment modality, evaluate disease recurrence, classify molecular subtypes, and treat cases.

As we all know, different pyroptosis-associated factors, like genes in gasdermin family, inflammatory vesicles, and proinflammatory factors, are tightly related to cancer genesis and metastasis. In this work, we firstly established the pyroptosis-associated profiles and found that pyroptosis was the main protective factor for the prognosis of melanoma patients. Then, after WGCNA, some key genes (*n* = 984) associated with pyroptosis-associated profiles were identified. Then, LASSO and stepwise regression analyses were performed to construct PPRS by incorporating 8 prognostic genes, among which, 7 (except GRIPAP1) were associated with cancer. HPDL (4-hydroxyphenylpyruvate dioxygenase-like protein) and FITM1 (interferon-induced transmembrane protein 1) are aberrantly expressed within different cancers (including pancreatic ductal adenocarcinoma (PDAC), non-small-cell lung cancer (NSCLC), and breast cancer), and they are related to cancer cell growth, migration, metabolism, and redox balance [36, 37]. Guanylate binding protein 2 (GBP2), a member of the GTPase family, is crucial to host immunity against pathogens and has been demonstrated as a potential immunotherapeutic target for multiple “cold” tumor, such as proficient-mismatch-repair or microsatellite stability (pMMR/MSS) colorectal cancer [38]. Besides, polymorphisms in FCGR2A have been validated with high degree association of trastuzumab and cetuximab benefit in the adjuvant treatment of breast cancer [39] and colorectal cancer [40]. Meanwhile, overmodulated expression of HAPLN3 was suggested to relate with the initiation of breast cancer [41]. TRIM34 expression in goblet cells plays an essential role in generating the inner mucus layer and preventing excessive colon inflammation and tumorigenesis [42]. However, four genes, i.e., HPDL, GRIPAP1, GBP2, and TRIM34, have been reported to exhibit prognostic significance with respect to melanoma for the first time, while HAPLN3, CCL8, FCGR2A, and IFITM1 [43–46] have been reported to relate with the initiation and immune microenvironment of cutaneous melanoma. Thus, it is necessary to conduct further in vitro and in vivo investigations to examine the role of those 4 pivotal genes in melanoma and their precise mechanisms of action.

According to GSEA, cell cycle activation along with immunoinflammatory response pathway suppression was the possible risk factor for PPRS-high samples. Based on the critical role of cancer stem cell-like cells (CSCs) on deregulation of cell cycle resulting in the abnormal proliferation and therapeutic (including both chemotherapy and immunotherapy) resistance [47], we explored the association between tumor stemness and our constructed PPRS. Our study showed that the HPDL and PPRS were associated positively with tumor stemness. These findings confirmed that our PPRS is a risk characteristic and that the HPDL might promote the proliferation of tumor cells and inhibit the differentiation of tumor stem cells through different pathways. However, the regulatory effect and mechanism of HPDL on the biological behavior of cancer stem cells have not been reported so far, which can be further studied and explored.

Besides, due to the important impact of pyroptosis on tumor immunity, we examined the different immune infiltration levels between low and high PPRS groups. According to recent reports, resting memory CD4 T-cells are mainly enriched into SKCM tissues. Further, among different cancer types, *γδ* T-cells are discovered in tertiary lymphoid structures, indicating that they are related to generating the constantly effective antitumor immunity [48]. M1 macrophages are related to antitumor immunity, whereas M2 macrophages are related to melanoma occurrence and invasion [49]. The present work suggested that high PPRS cases had decreased percentages of M1 macrophages and T-cells. Based on IPS analysis, high PPRS cases were associated with remarkably lower IPS scores, which suggested the low probability of applying ICIs among such patients.

PPRS displayed some advantages. (1) First of all, the prognosis model was accurate in prognosis prediction. As revealed by DCA, our pyroptosis-associated nomogram was accurate in the prognosis prediction relative to additional pathological features. (2) According to the risk score obtained by the model, SKCM cases were divided as 2 groups. In line with TIME and immune infiltration analyses, differences in several factors were significant between 2 groups, especially for checkpoint gene levels, which were consistent with differences obtained in enrichment analyses. Therefore, immunotherapies targeting the above 8 components in the PPRS can be utilized for cancer treatment. (3) Prior works constructing pyroptosis-related prognostic signatures just take into consideration those 27 known genes from the database for the direct regulation of pyroptosis; however, no attention is paid to the cascade reaction and interaction of such genes with others within cancer (the complex pathological process), resulting in the limitation of the constructed gene signatures. We adopted ssGSEA to quantify the characteristic scores of pyroptosis-associated profiles of each sample and acquired the coexpression genes related to the scores for constructing the PPRS. It considers the mutual regulation of the whole biological network; as a result, it can be used in different cancers.

Some limitations should be noted in our study. First, we obtained 679 cases from microarray and RNA-seq platforms, which indicated that our results were highly reliable, robustness, and without any platform bias. However, more prospective studies are warranted for verifying the effect of PPRS on evaluating the survival and immunotherapeutic response of SKCM. Second, our study was carried out among cancer patients from public databases according to bioinformatic analysis, and further research is necessary to verify those identified hallmarks among clinical samples. Third, the pyroptosis-related mechanism and immunoregulation of those 8 genes should be further investigated in SKCM cases. Finally, it is of great importance to analyze the alterations of pyroptosis features within cancer across the treated SKCM cases, as well as the impact on immunotherapeutic responses of cases.

## 5. Conclusions

We built an 8-PAG-based signature for predicting the survival of SKCM. The as-constructed signature was accurate in predicting prognosis of cutaneous melanoma. Besides, it accurately indicated tumor microenvironment and immune infiltration in patients and provided theoretical basis for clinical treatment. Therefore, our constructed signature is promising as a new diagnostic biomarker and therapeutic target.

## Data Availability

The dataset used in this study is available in GSE22153 (https://www.ncbi.nlm.nih.gov/geo/query/acc.cgi?acc=GSE22153), GSE54467 (https://www.ncbi.nlm.nih.gov/geo/query/acc.cgi?acc=GSE54467), and GSE65904 (https://www.ncbi.nlm.nih.gov/geo/query/acc.cgi?acc=GSE65904).

## Supplementary Materials

Supplementary Materials Figure S1: comparison of the PPRS with other known SKCM prognosis prediction models reported in literature. The ROC and KM curves of 4-gene signature (A), 12-gene signature (B), and 5-gene signature (C) are shown. (D) C-index (concordance index) was used to evaluate the predictive ability of the 4 models.
