# Supplementary material for: Identification of Pyroptosis-Relevant Signature in Tumor Immune Microenvironment and Prognosis in Skin Cutaneous Melanoma Using Network Analysis
Source: Stem Cells Int. 2023 Feb 8;2023:3827999. doi: 10.1155/2023/3827999 (PMC9931490; doi:10.1155/2023/3827999)
Supplement: Supplementary Materials — Figure S1: comparison of the PPRS with other known SKCM prognosis prediction models reported in literature. The ROC and KM curves of 4-gene signature (A), 12-gene signature (B), and 5-gene signature (C) are shown. (D) C-index (concordance index) was used to evaluate the predictive ability of the 4 models. [file 3827999.f1.pdf]

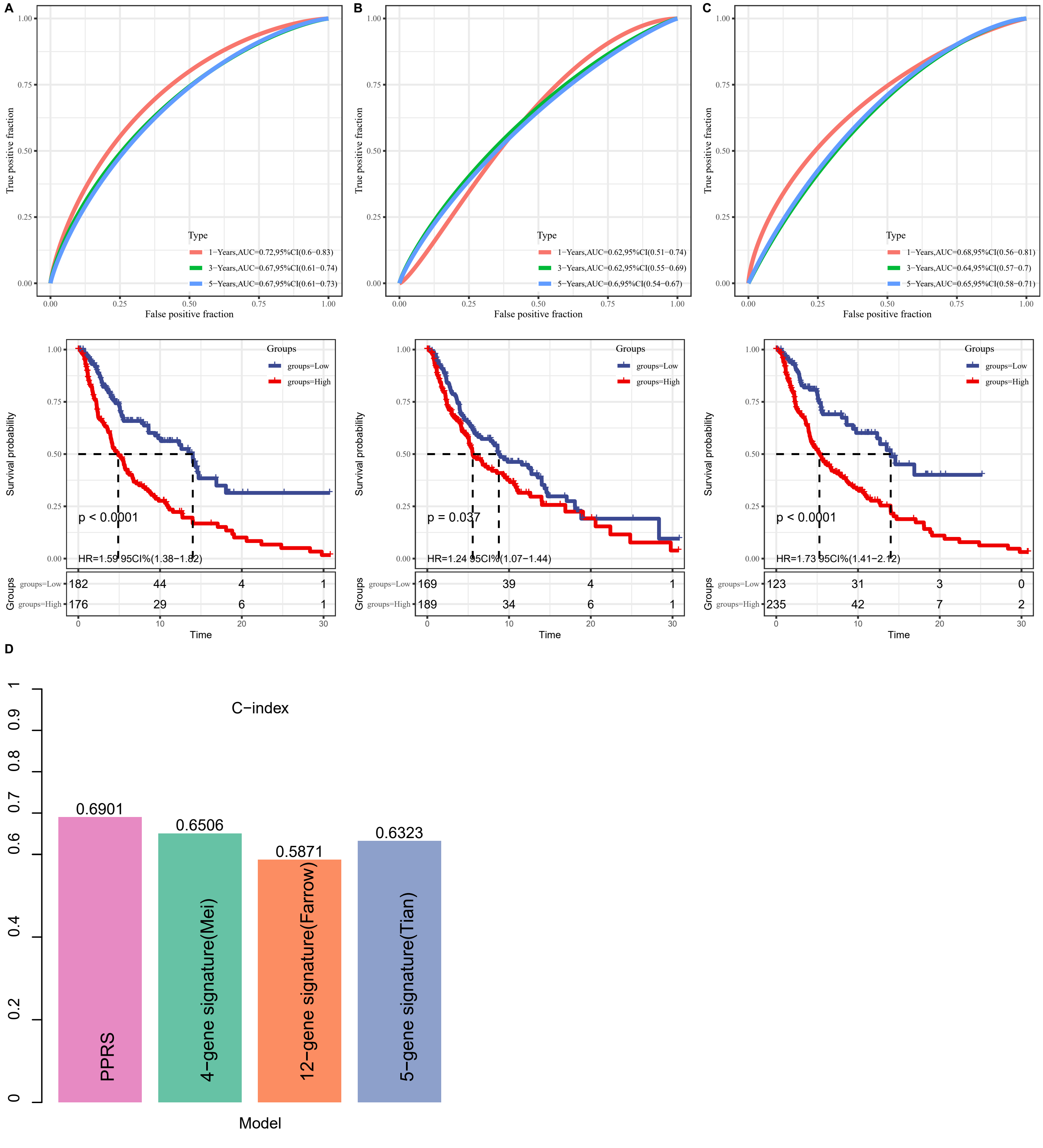

Fig.S1 Comparison of the PPRS with other known SKCM prognosis prediction models reported in literature. The ROC and KM curves of 4-gene signature (A), 12-gene signature (B) and 5-gene signature (C) are shown. (D) C-index (concordance index) was used to evaluate the predictive ability of the 4 models.
